# This is your brain on death: a comparative analysis of a near-death experience and subsequent 5-Methoxy-DMT experience

**DOI:** 10.3389/fpsyg.2023.1083361

**Published:** 2023-06-29

**Authors:** Pascal Michael, David Luke, Oliver Robinson

**Affiliations:** ^1^Centre for Mental Health, School of Human Sciences, Old Royal Naval College, University of Greenwich, London, United Kingdom; ^2^Centre for Psychedelic Research, Department of Brain Sciences, Faculty of Medicine, Imperial College, London, United Kingdom

**Keywords:** 5-MeO-DMT, endogenous, psychedelics, near-death experience, encephalitis, mystical experience, thematic analysis

## Abstract

**Introduction:**

Much research has focused on the modeling of the near-death experience (NDE) by classical and atypical psychedelics; however, to date, no study has reported on the relationship between the NDE and the experience induced by the highly potent, endogenous psychedelic drug 5-Methoxy-DMT (5MeO-DMT). This article presents a case study of an individual who is popularly documented to have had a profound near-death experience while in a coma caused by bacterial meningoencephalitis. Additionally, the individual also subsequently underwent an experience with 5MeO-DMT.

**Methods:**

A semi-structured interview was conducted with the subject concerning his experiences with both the NDE and 5MeO-DMT. A basic thematic analysis was performed on both the original text describing the NDE as well as the interview itself, which mainly focused on the subject's experience with 5MeO-DMT. This analysis was organized to identify both the similar and different emergent themes between the two states, with a particular emphasis on the subject's perceptions of the similarities and differences between the experiences.

**Results:**

There is a very high level of comparability between the original NDE and psychedelic experiences in general, including shared characteristics such as entering other worlds, meeting menacing or benevolent entities, experiencing synesthesia, perinatal regression, and lucid dreamlike properties. Much comparability was also identified with the 5MeO-DMT experience, in particular the major mystical experiential domains, such as ego dissolution, but especially transcendence of time and space. However, there were also a few unique themes (life review, the deceased, and the threshold) that emerged in the NDE that were not present in the 5MeO-DMT experience or other psychedelic experience studies, suggesting that these themes may be more unique to the NDE.

**Discussion:**

Despite such similarities, the participant asserted that his NDE and psychedelic experiences were not similar enough to be attributed to endogenous psychedelics. In this study, we discussed several mechanisms that could potentially account for the NDE, including lucid dreams and perinatal regression. However, the study also explored the possibility that the unique etiology of the participant's NDE, bacterial meningoencephalitis affecting the neocortex, may have triggered similar downstream neural activity as that initiated by psychedelic agents through pyramidal neuronal activation. This hypothesis is presented with appropriate caveats and acknowledged as speculative.

## Introduction

The case study presented in this article focuses on a 54-year-old Caucasian man from North America, who is popularly recognized (Alexander, 2012) and clinically documented (Khanna et al., 2018) for having had a near-death experience (NDE). The subject originally reported a score of 29 out of 32 on the Near-Death Experience Scale (NDES). The subject's NDE occurred during a 1-week coma period from 10th to 6th November 2008, caused by a high-fatality bacterial meningoencephalitis. During this time, the subject had Glasgow Coma Scale scores ranging from 6 to 11 (<9 = deep coma; further clinical details are reported in the study by Khanna et al. (2018). This article provides a systematic and comparative qualitative analysis of his NDE and subsequent experience with 5-methoxy-*N,N*-dimethyltryptamine (5-MeO-DMT), a potent endogenous psychedelic drug. While the primary focus was on the potential of this substance as a model of the NDE, this analysis also considers its possible role in inducing the NDR.

### 5-MeO-DMT

5-MeO-DMT (hereafter called 5MeO) is a fast-acting indoleamine that has the greatest affinity for the 5HT-1A site; it is found in the *yopo* snuff, which is derived from the Anadenanthera bean of the Amazonian basin and contains other compounds such as *N,N-*DMT and bufotenin. This drug is also present in the bufotoxin of the Bufo Alvarius toad found in the Sonoran desert (alongside bufotenin). In total, 15 studies have putatively identified the presence of 5-MeO-DMT in the urine, blood, and cerebrospinal fluid (CSF) in a subset of human subjects (Ermakova, 2023), and Smythies et al. (1979) and Corbett et al. (1978) detected it in CSF using more reliable GC-MS methods. In addition, Szabo et al. (2014) found that treatment of inflamed dendritic cells with 5 MeO resulted in suppression of pro-inflammatory cytokines and inflammatory T cell production, and elevated anti-inflammatory cytokines. Although the physiological roles of the substance are not yet known, these findings are suggestive of endogenous functions and clinical potential, at least in immunomodulation.

Recent studies have indicated that 5-MeO-DMT has the acute effect of reliably inducing mystical-type experiences and may have therapeutic applications that are highly comparable to the NDE. In fact, vaporized butotoxin, which contains 5-MeO-DMT, has been shown to induce “complete” mystical experiences in over 75% of individuals who use it. The intensity of these experiences is equivalent to reports of high-dose psilocybin use (Barsuglia et al., 2018a). Such mystical experiences from synthetic 5MeO, as well as the enduring effects of meaningfulness, spirituality and wellbeing, are also found to be significantly higher when conducted within a safe and supportive structured setting (Sepeda et al., 2019). This is echoed by the use of “benefit enhancement” strategies elevating acute mystical experiences and long-term sense of personal meaning and spiritual significance (Lancelotta and Davis, 2020).

Studies conducted in naturalistic settings across Europe have demonstrated improvements in life satisfaction, depression, and anxiety after individuals experienced 5MeO. These improvements were typically sustained for up to 4 weeks, most of which were found to be positively correlated with the level of ego dissolution experienced during the 5MeO experience (Uthaug et al., 2019). In this study, affect and non-judgement improved for at least 1 week after the experience and were positively correlated with the quality of the psychedelic experience. Moreover, salivary levels of cortisol and pro-inflammatory IL-6 were reduced after the experience (Uthaug et al., 2020). Furthermore, an online survey of users who used 5-MeO-DMT in structured group settings found that improvements in depression and anxiety were associated with a greater reported sense of enduring meaningfulness and spirituality (Davis et al., 2019). A case study on the enhancement in mood and cessation of alcohol use after 5MeO administration (albeit subsequent to Ibogaine administration) in an individual with alcohol abuse has also been reported (Barsuglia et al., 2018a) Here, these changes were associated with increased perfusion via PET imaging in brain regions related to substance disorders and classical psychedelic action (such as the caudate, putamen, insula, cerebellum, and temporo-occipital areas). Treatment of inflamed dendritic cells with 5 MeO suppressed pro-inflammatory cytokines and inflammatory T cell production and elevated anti-inflammatory cytokines (Szabo et al., 2014). Ermakova et al. (2022) produced a recent narrative review of research with 5-MeO-DMT, whereas Reckweg et al. (2022) reviewed the pharmacology, subjective effects, and therapeutic promise of the substance, including the three completed and eight ongoing clinical trials. The single published Phase 1 trial by Reckweg et al. (2021) identified significant increases after dose escalation from 2 to 18 mg 5-MeO-DMT in peak and mystical experiences, albeit seemingly without alteration in cognition and wellbeing.

### The rationale for the study

The pharmacologically analogous *N,N-*DMT (hereafter, DMT) experience has been rigorously associated with the NDE (Timmermann et al., 2018), as have many other classical or atypical psychedelics (ketamine: Corazza, 2008; Martial et al., 2019). Prior analyses of the DMT experience from a field study have referenced comparability with the NDE (Michael et al., 2021, 2023) and systematically compared the qualitative content of the DMT experience and the NDE [Michael et al., (in submission)]. However, no prior studies have examined the relationship between the 5-MeO-DMT experience and the NDE. The present article, therefore, aims to be the first to assess the convergence or divergence between 5-MeO-DMT and the NDE via comparative qualitative analyses, primarily psychometric analysis. They also discussed how this relates to the link between DMT and the NDE and which substance may simulate the experiential features of the NDE more closely. Other than Grof's (1994) report finding high comparability between LSD and a subsequent NDE, no other study has systematically reported on persons experiencing both a classical psychedelic and a near-death experience, as well as their personal reflections of comparability.

## Methods

### Recruitment

The first author of the present case study met the participant after their presentation at an academic conference (Beyond the Brain, 2018) and was informed of the participant's experience with 5-MeO-DMT. Upon asking if he was interested in taking part in a planned study to investigate the experiences of those with both near-death experiences and DMT or analogous experiences, he requested that this author contact his personal secretary. Participant information and a consent form were sent to the individual, and after acceptance, a video call was scheduled to conduct the interview.

### Procedure

A semi-structured interview was conducted with the participant to discuss the nature of his 5-MeO-DMT experiences and their comparability to his initial NDE. The interview took place on 13th November 2019, which was 22 months after the participant's psychedelic experience.

The interview duration was 1 h and 21 min. Interview questions commenced with “*Please describe in as much detail as possible your experience with 5-MeO-DMT*,” with subsequent probing questions including, for instance, “*Was there any kind of sensorial or visual experiential structure as well [in your 5-MeO]?*” “*Can I ask for an elaboration of what you refer to as the ‘counterfactual'...?*” “*I [also] wonder what your comments would be in terms of the threshold of no return that comes up with NDEs?*” A more comprehensive list of questions can be found in the Supplementary material.

The interviewee reported having three experiences with venom from the Sonoran Desert Toad that were administered via a glass pipe. The doses escalated over time, with the experience deemed by him to be “the most significant” being focused on during the interview. The interviewee also reported experiencing highly “similar but more powerful effects at (sic) higher dose.” The date of this chosen experience was 21st January 2018, and the dose was 46 mg. The setting was described as “In a comfortable private home setting with close friends.”

### Analysis

A thematic analysis (Braun and Clarke, 2006) was performed on the published NDE account, as reported by the participant in a popular book (Alexander, 2012), as well as on the novel interview focusing on the latter 5-MeO-DMT experience. This was conducted using Microsoft Word, entailing the listing of many specific codes, which were subsequently collated into broader final theme headings, with this process being completed first with the NDE and then with the 5-MeO-DMT experience. The analysis was inductive, deriving themes purely from transcript data. Finally, identifications of each theme's presence in either one of the experiences or both lead to their final categorization as either similar or different.

The participant was also asked to provide an indication on a given scale of how similar the experiences appeared, and the NDE Scale (Greyson, 1983) was also administered, with the participant answering regarding his experience with 5MeO and, separately, his near-death experience. This quantitative add-on enabled the provision of a structured comparison of the generic, phenomenological structure as a complement to the richer qualitative analyses.

### Ethics

The present study, including the interview with the participant who experienced the NDE, was approved by the University of Greenwich Research Ethics Committee (Ref. 18.5.5.17).

## Results

### Qualitative analysis

**Figure 1:**
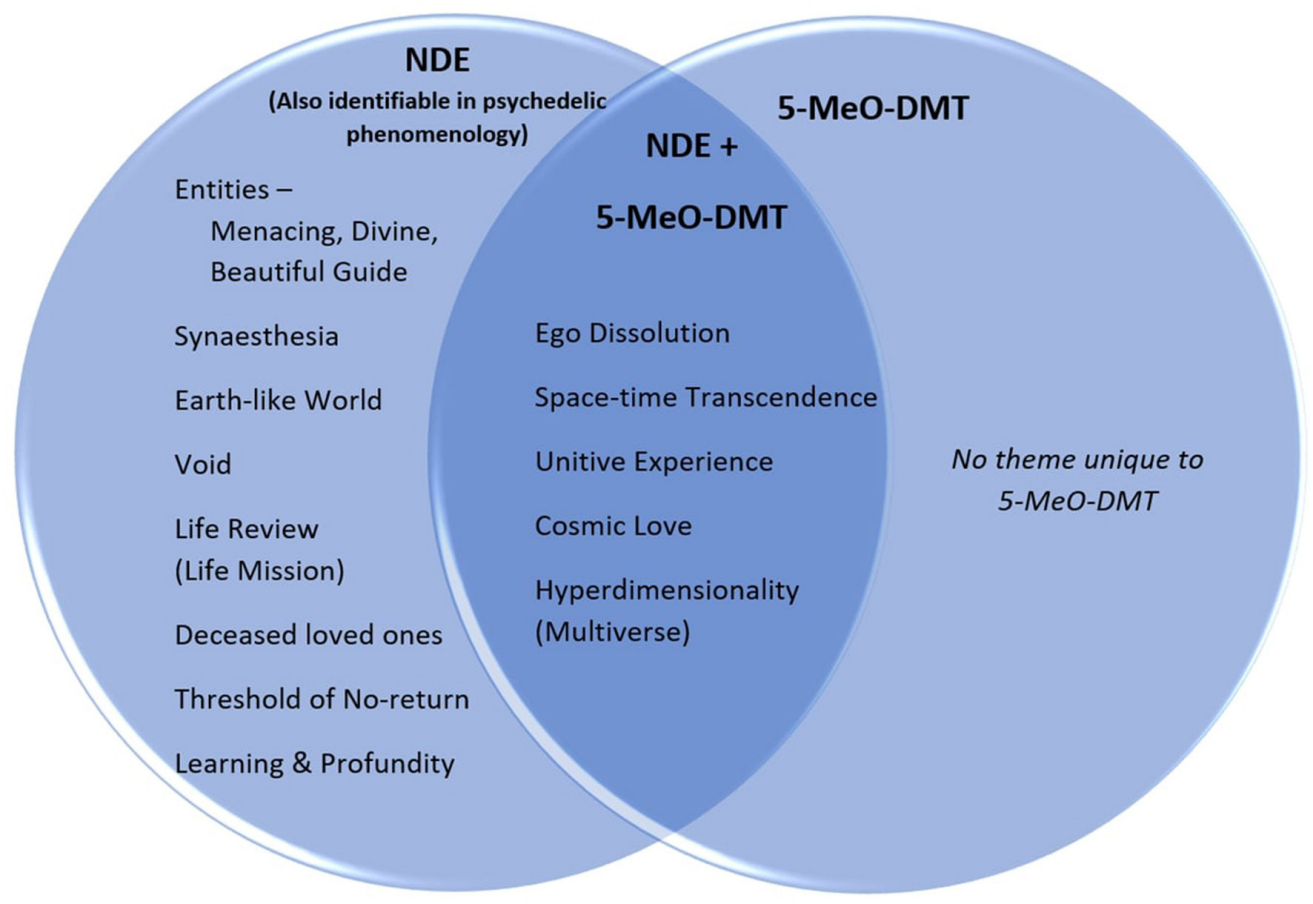
Themes present in either the near-death experience, or 5-MeO-DMT experience reported by *Nikoli*, or their overlap. All features of 5-MeO-DMT experience also present in NDE experience.

This analysis section is divided first into similar and later into different sets of emergent themes between the experiences, where every theme is accompanied by exemplary quotes from both the NDE and 5MeO (inclusions of “(?)” within quoted excerpts denote the partial audibility of the previous word). A diagrammatic summary of the themes extant in both states and their overlap is given in Figure 1. Importantly, given the unique ability of the experiment to comment from a first-person perspective on both states, their personal perceptions regarding similarities and differences are diffusely expressed. Finally, narrative commentary regarding the experiences is also integrated throughout (mainly in the section pertaining to *differences*, comparing given NDE excerpts to psychedelic phenomenology). In the continuation of the qualitative analysis, after the quantitative description, this commentary eventually encompasses other possible, not necessarily mutually exclusive, mechanisms of this and other NDEs. A particular focus on the said mechanisms will help the present study take a more neurophenomenological orientation, examining how the unique etiology of this case and potentially similar cases may mirror and produce comparable effects to how psychedelics dysregulate higher cortical networks and may, as such, echo the neural and computational action of psychedelics.

### Similarities

#### Ego dissolution

##### Near-death experience—Original text (hereafter, “NDE”)

Early in his NDE report, *Nikoli* recounts that his state of consciousness “was without memory or identity—like a dream… [I] was a lone point of awareness.” Later, he explains

“I had no real center of consciousness. I didn't know who or what I was or even *if* I was. I was simply… there, a singular awareness in the midst of a soupy, dark, muddy nothingness… most [other NDErs] remembered their earthly identities while away from their earthly forms… They were aware that their living relatives were still [on earth]… met friends and relatives who had died before them… Many… have reported engaging in life reviews… I experienced none of these events… How could I… not realize that on earth I was a doctor, husband and father?... I was in a position similar to that of someone with partial but beneficial amnesia. That is, a person who has forgotten some key aspect about him or herself, but who benefits from having forgotten it… It allowed me to go deep… without having to worry about what I was leaving behind… I had come from nowhere and had no history, so I fully accepted my circumstances… And because I so completely forgot my mortal identity, I was granted full access to the true cosmic being I really am (and we all are).”

What is articulated throughout here as “partial but beneficial amnesia” is virtually identical to what is referred to in the psychedelic sphere as “ego death.” That is, the annihilation, though temporary, of the sense of one's individuated self and all of its concomitant autobiographical memories often gives rise to an experience of being a “cosmic being.” The benefits of this are made abundantly clear by the great number of publications on the psychedelic-induced mystical experience, with the dissolution of ego as a key dimension and being the primary predictor of therapeutic or other advantageous effects (e.g., Griffiths et al., 2008; Barsuglia et al., 2018b; Haijen et al., 2018; Roseman et al., 2018; Kettner et al., 2019).

##### 5MeO experience—Interview, including comparison (hereafter, “5MeO”)

*Nikoli* describes that in his NDE, particularly in the context of comparing its *similarity* with the 5MeO episode, “my ego-mind was gone… [only] that inner observer, the neutral observer… the voice in your head, our little ego-mind, is not who we are and is not our consciousness… [it] is little more than a parlor trick, pay it no mind… that awareness within…is the part of us which expands tremendously when liberated from the shackles of brain and body at physical death.”

#### Time or space-time transcendence

Despite the significant similarities in terms of time transcendence in the excerpts below, there is a dissimilarity in that we see the description of “geometric patterns,” further qualified as transforming, evolving, and inter-locking, as well as an emphasis on the mechanistic details of how time works, which were characteristic of his 5MeO experience and *not* the NDE:

##### NDE

“I (whatever “I” was) had always been there and would always continue to be.”

“…the vagaries of time in these worlds beyond… continued to hold… ponder how time lays itself out in dreams… “before” or “after” become tricky designations. You can be in one part of a dream and know what's coming, even if you haven't experienced it yet.”

##### 5MeO

“…the 5MeO was [more] profoundly, richly imbued with the witnessing of the interleaving of time and space and how they're really one. And this incredible… seeing [of] the interleaving, it was almost like you could see all the various elements of space time and all the possible permutations, and they simply seemed to come into a locking fashion. I could watch the whole thing evolve… I could really see those geometric patterns that included what I call *counterfactuals*, in other words the possibilities that were there for a choice but that my will as a higher soul rejected. But I could still see them, I could see the possibilities… pathways of actualities.”

“…you could basically see cause and effect over time and across space, but you can see them all ‘at once'. In many ways its reminiscent of… [the] life review situation [which, strictly, *Nikoli's* NDE did not include]… It's showing us our notion of the linear flow of time in these bodies in 4D space-time is in so many ways fabricated, it's there as a narrative to lay down a pathway that sets a stage for us to face life's circumstances… and for me the 5MeO gave me a glimpse of how all of that works. It was like having a microscope in my [NDE's] *Core* realm journey and being able to look at why things appeared the way they did in my NDE, and see the mechanism of it. And the mechanism wasn't as apparent during the NDE, just all of the lessons, all the flow, the relationships, the big picture was very clear. But if anything, the 5MeO, what it gave me in addition is that ability to witness that interleaving—like all of these very fancy tiles that would come together and self-form each other in this evolving set of patterns… it was much more micro-focused on the interleaving of time and space.”

Indeed, complex fractal imagery in flux, providing an apparent window into the mechanics of the universe, is a classic feature of psychedelic phenomenology, especially in the case of *N,N-*DMT (Michael et al., 2021). However, his NDE account does report other stereotypical psychedelic visual motifs, as he and a guide “were riding along together on an intricately patterned surface, alive with indescribable and living colors—the wing of a butterfly… all of the [butterflies] together…were a river of life and color moving through the air.”

*Nikoli* then elaborates on this overlap in the experience of time, referring to its implications in his NDE for reincarnation:

“[The realm in the NDE] with earth-like features but also deep spiritual features, had also what I call ‘deep time'… [this] has to do with the much bigger ordering of progression of our souls and evolution of all consciousness, and that's what's occurring in that level, that *Gateway* valley… an ordering of things like reincarnation, where we improve ourselves with every incarnation, going through the life review, then planning the next set of challenges for the next incarnation.”

“It was a very visual experience, the way I saw it deep in coma… it was a tapestry, and the word that comes to mind is Indra's net. But it was this beautiful tapestry of interwoven silver and golden fibers that represented lifelines of higher soul journeys. And I saw this rich interweaving in life reviews and how that was part of the metallic garnishing at the highest points of those peaks of the weaving of those life reviews. Another way it was presented to me was this absolutely glorious… flying vision, and I could see the fish down under water, that was our material realm, going under the illusion of 4D and Earth time, and then popping up out of the waters when our higher soul leaves the body, reunites with higher souls up in the air above the water, all in this much greater illumination of what's going on, and trading of information—then *boom*, diving back into the water again for the next incarnation.”

Although the 5Meo experience featured geometric arrays of time space that were absent in the NDE, the NDE still included clear visual representations of lifelines, which were described as “tapestry-like” and composed of fibers of light. Moreover, while visually symbolic imagery such as the flying-fish metaphor was absent in the 5MeO experience, the NDE featured archetypal symbolism, such as the lifelines, that is characteristic of deep psychedelic journeys.

#### Unitive experience

The picture he offers below of his NDE is heavily redolent of an *in-utero* regressive experience, discussed directly in the below segment on potential mechanisms:

##### NDE

“…there was no difference between “me” and the… half-familiar element that surrounded me. But this… boundary less immersion gave way to… feeling like I was… trapped in it.”

Nevertheless, he expounds on this to evoke a profound unitive experience and realizations as to the deeply intertwined nature of things, which he expresses to be a fundamental reflection of his 5MeO experience:

“Everything was distinct, yet everything was also a part of everything else, like the rich and intermingled designs on a Persian carpet [again, evoking the ‘tapestry' not unlike the geometry of the 5MeO, or psychedelic experiences generally]… The world of time and space in which we move… is tightly and intricately meshed within these higher worlds… all worlds are part of the same overarching divine Reality. From those higher worlds one could access any time or place in our world.”

##### 5MeO

“…the 5MeO, if I had to put it on a scale, was much more aligned with the deepest aspects of the NDE in terms of the oneness… [It was] a very strong…unification, the oneness of dualism… the [5MeO] DMT certainly had the quality of bringing those dualities together, so you realize they're just a spectrum, and the polarizations in essence didn't exist in their own right.”

He continues:

“…*the Core realm [of the NDE], its oneness, the origin of all experience and the cross-over of the awareness of the universe and the universe itself—to me in all of my psychedelic experiences… the thing that most matches up with that is the 5MeO* [Italics mine].”

However, *Nikoli* caveats this primary parallel with a difference in degree: “[The 5MeO was] like looking through a little peephole, as opposed to being full-bore swimming and being immersed in the Pacific ocean of being completely into that oneness experience [of the NDE].”

The following excerpt about his NDE demonstrates the concept of “anthogenesis” (generating the divine within), in which one experiences an equivalence between their own consciousness and that of the divine, where “entheogen” is technically another denotation for psychedelic medicines, which *Nikoli* states is tantamount to love itself:

“Oneness with God, in that *Core* realm, my awareness, higher soul experience, was one of becoming identical with that God force, in terms of creative possibilities… conscious awareness at its root is that God force, that so many experience in an NDE as a force of pure love and pure wholeness and healing.”

#### Cosmic love

This love, of more divine quality, is also extended to an encounter *Nikoli* shares with a girl whom he does not recognize:

##### NDE

“She looked at me… It was not a romantic look. It was not a look of friendship. It was… somehow beyond… all the different types of love we have down on earth. It was something higher, holding all these other kinds of love within itself.”

*He goes on to* elaborate on his encounter with her in one realm, then with an orb, identifying it as the same girl, in yet another realm in which “Om,” the Source of all things, resided. The primacy of love was once again a core communication:

“Through the Orb, Om told me that there is not one universe but many—in fact, more than I could conceive—but that love lay at the center of them all… in the larger picture love was overwhelmingly dominant, and it would ultimately be triumphant.”

“If I had to boil [the message] down further, to just one word, it would (of course) be, simply: *Love*. In its purest and most powerful form, this love is… *unconditional*. This is the reality of realities, the incomprehensibly glorious truth of truths that lives and breathes at the core of everything that exists.”

*Nikoli* explicitly points out the consistency between NDEs and psychedelics in general in the following quote in terms of the profound love that can be felt, which is itself inextricably intertwined with the sense of unity discussed above (and in the final quote). Interestingly, however, one difference he alludes to here is that the NDE appears more personal, illustrating the content of one's life (such as via the review), compared to the 5MeO, which may have been more transpersonal, conveying the mechanisms transcending immanent reality:

##### 5MeO

“…one [can] witness the bigger picture [in] an NDE [vs 5MeO]; how one's life had unfolded and feel the emotional power of one's actions and thoughts on others because that's another hallmark of the life review… you experience it more from the emotional viewpoint of those around you… And that's why the life review is so important for course-correction between lives—you're more treating the golden rule as it's meant to be part of the rule of the universe, to treat others with love. *And the one thing NDErs see and is certainly common in psychedelic experiences, is that sense of love and connectedness, and we start to feel intimately part of each other. To me that's an important lesson from NDEs that also comes into this world via many psychedelic experience* [Italics added].”

We receive glimpses in this final one of themes to be expounded on below; those of hyperdimensionality, as well as experiences of light, a sense of being taught, and a communicating guide. The latter three do not seem to be particularly inherent, the guide especially so, to the 5MeO trip (albeit these are certainly well-known to occur in the psychedelic space):

“…becoming one with that love force, and kind of leaving all the dualities behind, that was that *Core* realm… in that core realm I had that *Oversphere*, this higher-dimensional multiverse as a kind of teaching tool, and this sense of this brilliant light brighter than a million stars, and an interpreter or a translator, and then all of this happening in an infinite realm that was overflowing with unconditional love. Again that's something… I think people can get through… the psychedelic experience, this more loving and connectedness.”

#### Hyperdimensionality, multiverse

In the 5-MeO, as we see in the following, we not only comparably gleam a sense of universal plurality as in the NDE, but also the shared themes of unity, as well as receiving teachings from and about the cosmos:

##### NDE

“I saw the abundance of life throughout the countless universes, including some whose intelligence was advanced far beyond that of humanity. I saw there are countless higher dimensions.”

##### 5MeO

“...our minds can simultaneously experience much bigger swathes of time and space. It points to my NDE, the entire higher dimensional multiverse throughout infinite dimensional space in all of eternity and infinity, was this tiny little *Oversphere*, there as an instructive tool in the setting of pure oneness with the divine.”

Also identified as consistent between the two states was the professing of ineffability.

### Differences

The following excerpts for each theme are either singularly from *Nikoli's* NDE report (appearing in roughly chronological order) or from the interview comparing the NDE to his 5MeO experience. Therefore, they are not directly contrasted as above, as they do not have counterparts in the other experience. Every theme heading here highlights the theme's presence in the *near-death experience* rather than in the 5MeO experience, illustrating the apparent differences between the two. However, references to other *N,N-*DMT experiences or other relevant literature are made to demonstrate the potential for the quoted near-death content to still be present in the realm of psychedelic phenomenology, even if not specifically in the context of 5MeO experiences.

#### Menacing entities (only in NDE, as with all the following headings)

Certain encounters with entities of a more hostile disposition were reported in his NDE rather than the 5MeO experience (as with all the following themes in this section):

“Grotesque animal faces bubbled up out of the muck, groaned… rhythmic chants… terrifying and weirdly familiar… The more I began to feel like a ‘me'… the more the faces… became ugly and threatening… movement around me became less visual and more tactile, as if reptilian, wormlike creatures were crowding past.”

In a detailed content analysis of the *N,N-*DMT experience from an observational field study, Michael et al. (2021) discovered that 8% of the 36 participants reported encountering “fearsome” beings, and another 8% reported encountering reptilian entities. Participant *LR*, specifically, reported a tactile-visual experience of “cosmic centipede”-like creatures “crawling around” him.

#### Synesthesia

Experiences that reflected the blending of the normally separate sensory modalities were a vivid element of *Nikoli's* NDE (also evocative of the sense of unity):

“It radiated fine filaments of white-gold light… the darkness around me began to splinter… I heard… a living sound, like the richest, most complex, most beautiful piece of music.”

“…flocks of transparent orbs … a glorious chant, came down from… the winged beings… The sound was palpable and almost material, like a rain that you can feel on your skin… Seeing and hearing were not separate… I could hear the visual beauty of the silvery bodies of those scintillating beings above, and I could see the surging, joyful perfection of what they sang… you could not look or listen to anything in this world without becoming a part of it.”

The description of synesthetic, scintillating orbs is remarkably similar to Kastrup's experience in an “altered state” (which, though not explicitly divulged, is classically DMT-esque and reminiscent of McKenna's lectures on DMT), as quoted in Kripal (2020). Katrup reported experiencing a “geometric world which expresses information through Christmas ball-like globes, or ‘Kandinsky scintilla' (referring to the abstract artist Kandinsky, who was known to be synesthetic). Synesthesia, particularly audio-visual, has also recently been shown to be very common with psychedelics, especially LSD (Luke et al., 2022).”

#### Divine being

In this study, we observed a highly synesthetic quality that seemed to bind together visual, tactile, auditory, and emotional components. Additionally, the text also conveyed a sense of the “omnipresence” of a powerful being, as well as some instances of “telepathic communication:”

“A divine breeze… shifting the world around me into an even higher octave, a higher vibration… I began wordlessly putting questions to this wind—and to the divine being I sensed at work behind or within it… the answer came instantly in an explosion of light, color, love and beauty that blew through me like a crashing wave… They answered [me], but in way that bypassed language… I was able to instantly and effortlessly understand.”

These features were also identified in 14% (synesthesia) and 36% (omnipresence) of Michael et al.'s (2021) DMT interviews from the aforementioned naturalistic study.

#### Earth-like world

The other world that *Nikoli* entered during his NDE elicited imagery of a utopian and arcadian earth:

“Below me there was countryside. It was green, lush… It was earth… but at the same time it wasn't… I was flying, passing over trees and fields, streams and waterfalls, and… people… children, too, laughing and playing… [They] sang and danced… I'd see a dog running and jumping among them… They wore simple yet beautiful clothes… the colors… had the same kind of living warmth as the trees and flowers.”

Michael et al.'s (2021) study found that 11% of DMT participants described “natural landscapes,” which were often described as “earthly yet divinized mirror-images of the earth” (Shushan, 2018). The participants' *EM* and *RH*, in particular, described the landscapes as “just like one of those… ancient Babylonian gardens” and “a garden of extraordinary beauty.” Furthermore, as with *Nikoli*, they also expressed an animistic and life-infused quality to the physical environment.

#### Beautiful guide

We will revisit an encounter with the girl in the NDE, who manifested in the form of an orb and provided support to *Nikoli* during his journey, emitting a loving energy:

“Someone was next to me: a beautiful girl with high cheek-bones and deep blue eyes… The girl's outfit… had the same overwhelming, super-vivid aliveness… Without using any words, she spoke to me [again, conveying telepathic communication]… and I instantly understood that it was true… The message had three parts: “You are loved and cherished, dearly, forever. You have nothing to fear. There is nothing you can do wrong.”

“I had the Orb as my companion… [which] was a kind of “interpreter” between me and this extraordinary presence surrounding me… the Orb [who in fact was (the girl on the butterfly-wing)] was guiding me.”

“…the “voice” of this Being was warm and… personal… It knew me deeply, and overflowed with… compassion, pathos… even irony and humor.”

Similarly, the majority of the beings encountered in Michael et al.'s (2021) report were also “enchanting” (56%), often “benevolent” (28%), and a major role served was also as a “guide” (14%). The function of these entities as “teachers” (25%), who were also “familiar” (28%), is similarly echoed in *Nikoli's* portrayal of his companion.

#### Void

An emergence into a space that seemed at once like an abyssal emptiness and yet suffused with nurturing light eventually occurred:

“…an immense void, completely dark, infinite in size, yet also infinitely comforting. Pitch black as it was, it was also brimming over with light… “Om” was the sound I remembered hearing associated with that omnipotent, omniscient and unconditionally loving God.”

Moreover, at least 14% of Michael et al.'s (2021) accounts mention such a “void”-like environment as this. More saliently, though, in another series of experiments involving both DMT and NDEs [Michael et al. (in review)]. This “Dazzling Dark” environment is mentioned in *Nikoli's* book description; it is a term originally used by Wren Lewis (1995) to describe his own NDE in awakening from “a vast blackness that was somehow radiant, a kind of infinitely concentrated aliveness or pure consciousness.” It is closely reminiscent of *DA's* change experience (*N,N-*DMT mixed with MAOI-containing herbs): “everything went black, but still shimmering and vibrating… this sensation that I was in space… yet at the same time that it was the same thing as the white light.”

#### Life review (life mission), deceased loved ones

An evaluation of one's life, as described in *Nikoli*'s NDE, is here connected to the moment of meeting deceased loved ones, which highlights the importance of relationships. These themes are not typically reported as being intrinsic to psychedelic experiences, according to Nikoli:

“…encountering souls of departed loved ones or profound life reviews where you realize the life review's really not about you but about your inter-relationships with others—those are the pieces that in general seem to be shallow in psychedelic drug experiences, especially the parts that line up more with one's life mission.”

#### Threshold of no-return

*Nikoli* identified what he “would call that ‘threshold' with what he ‘saw between the *Gateway* valley, which had a tremendous number of earth-like features.' also qualifying that that would be where a life review would happen, reunited with the souls of departed loves.”

Regarding these former two themes (life review and deceased), associated with the latter (threshold), the conjecture regarding their being shallow with psychedelics has been quantitatively supported. For instance, Greyson (2014) demonstrated that the features of seeing the deceased, having a life review, and coming to a border were characteristic of NDEs when compared to a measure of mystical experience. This is mirrored in Michael et al.'s (in review) finding that these same three items scored the lowest when the NDE scale was applied to DMT experiences. This, however, does not mean they are absent. For example, Michael et al. (in review) also include the life review and encountering the dead in 6% of DMT experiences each. This being so, the threshold was not exhibited in Michael et al. (in review), suggesting it as an especially unique symbol to the near-death experience, given its obvious symbolic denotation of the irreversibility of death. However, again in Michael et al.'s (in review) series of DMT & NDE experiments, *SR* reports in his change experience a “metal gate with gaps” that he was prevented from going through by a feminine entity as if signaling “you're not ready yet.”

#### Learning, profundity

As mentioned above, in the quote below, *Nikoli* again asserts a possible distinction between NDEs delivering more personal insights and more transpersonal content being the domain of psychedelics. Most saliently, though, *he* here alludes to the real possibility that it is the artifact of their having limited psychedelic exposure that may be the reason for opining in this way (and by extension, about other differences):

“I gave up drinking in ‘91, I'm a recovering alcoholic, the fact I was adopted so I spent a lot of my life thinking at some deep level, not consciously, that I wasn't worthy of life because my birth mother left me behind when I was 11 days old. So those deep knowings of oneness with the universe, of why that addiction would be there in the first place, all that stuff was very apparent through the wisdom of the NDE [compared to the 5MeO]… the 5MeO after my NDE… definitely taught me some interesting factors about the nature of reality, but whether they offered me any of the really deep knowledge of my personal journey, my higher soul's work with this universe, to me it's not so apparent. *Maybe if I had a more dedicated programme and I continued using them, then that would be one thing* [Italics mine].”

This idea of making these distinctions, such as revelations into one's personal tribulations, being related to limited awareness of psychedelics is especially born out in his seemingly not being cognizant of psychedelics' therapeutic effectivity in trauma and addiction. Specifically, research suggests this is not only due to neurobiological interactions but also subjective experiences, such as insights and life reviews (Schenberg et al., 2017; Wolff et al., 2019; Davis et al., 2021).

Also identified as inconsistent between the experiences was the special emphasis on hyper-reality in the NDE. However, this is an almost ubiquitous appraisal of the DMT space.

### Descriptive quantitative analysis

To quantify the comparability of the participant's subjective appraisal of the two states, *Nikoli*'s response to the following two questions suggested *low* similarity, underscoring the many qualitative differences explored above. However, this is despite the profound convergences observed in several domains:

i) How similar was your 5MeO-DMT and near-death experience? (1 = completely different; 10 = identical).
Answer: 2.
ii) What is the extent of your belief (if any) in the production/occasioning of your NDE being due to endogenous psychedelic-like brain chemicals, such as 5MeO-DMT (1 = absolutely impossible; 10 = absolutely definite).
Answer: 2.

**Figure 2:**
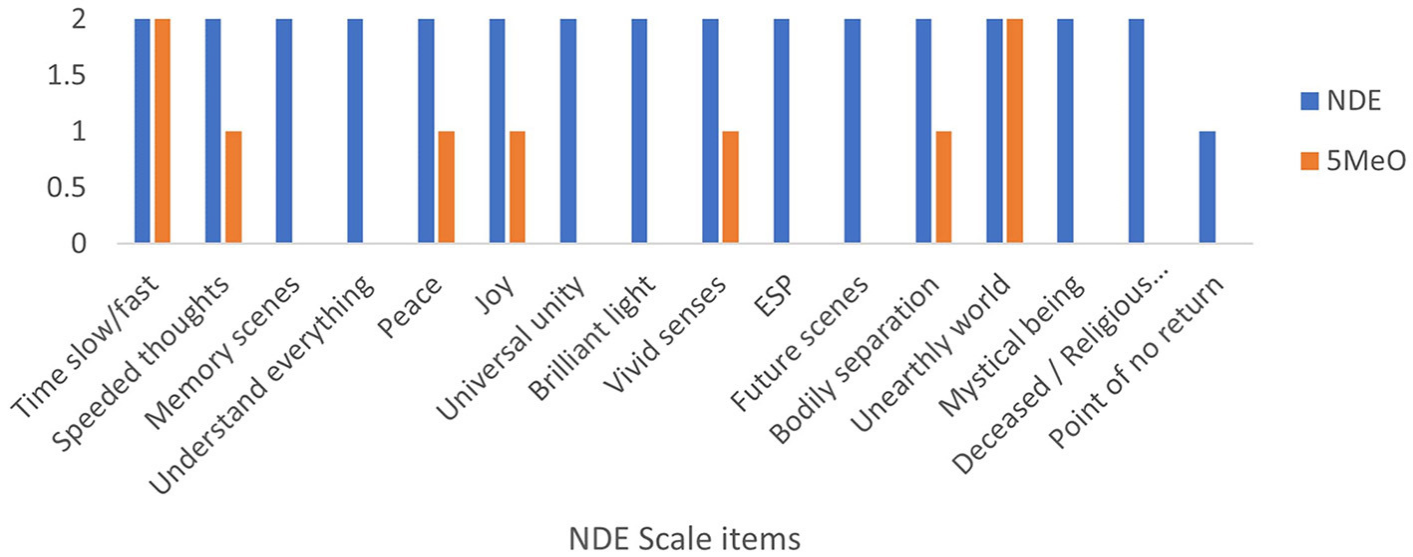
Scores from the near-death experience scale compared between the 5MeO-DMT and near-death experience.

From Figure 2, it can be seen that the NDE was rated highest for virtually all scale items (resulting in a total of 31/32), excluding the “threshold of no return,” which was only present to a certain degree. The psychedelic experience from the 5MeO-DMT, however, was rated substantially lower on almost all features (scoring only 9/32 in total). The emergence into some other realm and distortion in time were scored maximally, with separating from the body and the only other clearly mystical feature of peace/joy being also partially present (along with enhancements in thoughts and senses). In short, it might be concluded, though from this singular case study, that the 5MeO state is only a poor model for the NDE;it simulates it in only a small cluster of mostly shallow ways.

These psychometric results align somewhat with the wealth of interview data examined above, such as the elaborate descriptions of the transcendence of time taking place within a space entirely distinct from waking reality and with an emotional valence of apparently total positivity transpiring in both of the two states. However, from the qualitative analyses, it should be readily observable that *Nikoli*'s 5MeO experience did, at least in part, also consist of a sense of receiving insights and indications of the experient's personal life trajectories, such as the “counterfactuals” mentioned, but especially, the lack of endorsement of universal unity is disjointed with the qualitative report. While the credibility of the participant is not being questioned, these discontinuities could suggest the superiority of such in-depth semi-structured interviews in extracting richer, and thus more complete (and possibly more accurate) experiential data. Although quantitative scales can provide valuable insights, they have inherent limitations due to their allowing only fixed-answer responses with possibly ambiguous labels and descriptors. In this case, this may have incurred an over-estimation of the differences between two experiences. This, perhaps, is indicative of the strength of mixed methods results for necessary mutual complementation.

However, there are various similarities between the quantitative and qualitative results. For instance, bot reports did not mention the experiences of light and ESP, demonstrating significant convergent by feature absence. However, the NDE included evidence of other beings or religious/deceased spirits, while the 5MeO experience did not. These divergences may represent the most robust differences between the two states, and after accounting for possible discrepancies in quantitative scoring, they may be considered the most important distinguishing features. When considering such findings in the context of the literature, the experience of brilliant light is often strongly associated with the 5MeO experience, although it was not found (during scoring or interview) in the present study. The lack of other presences during the 5MeO experience, however, is particularly well-substantiated by existing extant data, as the phenomenology of this psychedelic is typically characterized by a non-dual and “contentless” nature, which differs from entity encounters classically transpiring during NDEs, where meetings with the dead are one of the commonest features (Charland-Verville et al., 2014).

### Qualitative analysis continued: potential mechanisms

The following analysis uses quotes from both the NDE account and the subsequent interview to explore potential mechanisms for the emergence of certain aspects of *Nikoli's*, as well as other individuals' NDEs.

#### Psychedelics, lucid dreaming, and mind-manifestation

While the focus of this article is on evaluating the 5MeO drug experience, particularly as a useful model for NDEs, the term “psychedelic” refers to the act of “manifesting of the mind.” Sensory experiences are determined by both psychical content and the dissolution of boundaries between one's outer phenomenal world and inner experience, and this highlights the relevance of dreaming in relation to NDEs. On two occasions, *Nikoli* has already compared his NDE to a dream; however, it is especially pertinent to consider lucid dreaming, wherein the dreamer is not only aware that he is dreaming but can manipulate their mind-constructed environments. *Nikoli* himself is aware of the connection between this phenomenon and NDEs: “…those who've become good at lucid dreaming, that's a little more the kind of engagement you get with the universe in an NDE.” However, as you can see below, he does not comment on how his own NDE reports are compellingly resounding of lucid dreamlike behavior and could suggest shared mechanisms between the two. Importantly, the endogenous psychedelic model is not incompatible with a lucid-dream view, as indeed, serotonergic psychedelics are known to simulate lucid dream content (Sanz and Tagliazucchi, 2018):

“I felt a sense of sadness unlike any I'd ever known. Emotions are different up there… deeper, more spacious—they're not just inside but outside as well. Imagine that every time your mood changed here on earth, the weather changed instantly… That your tears would bring on a torrential downpour, and your joy would make the clouds instantly disappear… how much more vast and consequential changes of mood feel like up there, how strangely and powerfully what we think of as “inside” and “outside” don't really exist at all. So it was that I, heartbroken, now sank into… an actual sinking.”

“I had to learn to navigate it on my own, which I did by acknowledging those musical melodies that could conjure up various portals between levels.”

“I actually had some control over my course… I was no longer trapped in this lower world. *With concerted effort, I could move back up to the higher planes…* I found myself wishing for the *Spinning Melody* to return… the gorgeous music, and the spinning ball of light emitting it, blossomed into my awareness… and I began to rise… *to know and be able to think of something is all one needs in order to move toward it…* [Italics mine] I accomplished this back-and-forth movement from the… *Earthworm's Eye-view*… to the *Gateway* and [then] to… the *Core* any number of times.”

Interestingly, the “spinning melody” in *Nikoli's* experience, which involved a highly synesthetic orchestration of both sound and light that he used to “move” between realms, has similarities to the experiences of those with depression who have reported experiencing “up-lifting,” healing, and novel music in their lucid dreams (Sackwild and Stumbrys, 2021).

#### Perinatal regression

The reactivation of pre-, post-natal, or *in-utero* memories has been proposed by Grof (1985, 2019) as a framework for certain psychedelic experiences, including the “basic perinatal matrices” 1–4, which have also been theorized as a model for NDE production. The following several excerpts are not only exceptionally uncanny in terms of their evocation of the experience from the perspective of a fetus but are explicitly articulated using such perinatal terminology, yet this possible explanation is not mentioned. Therefore, the endogenous psychedelic model and a perinatal regression explanation for NDEs are not mutually exclusive, as both can be conducive to such regressions:

In his NDE, *Nikoli* first found himself in an “underworld,” which he later characterizes as the “Earthworm's Eye-view,” composed of “visible darkness… Transparent… blurry, claustrophobic, suffocating.” A “deep, rhythmic pounding” also accompanied him, which was “*like a heart-beat…* as if a giant, subterranean blacksmith is pounding an anvil.” This space was primitive in nature, “*as if I had regressed back to…the very beginnings of life*.” He also recalls “conceptualizing that I might or might not survive.” Eventually he witnesses something “like roots, and *a little like blood-vessels in a vast, muddy womb*. Glowing a dark, dirty red… in a timeless red-brown sea” [Italics added].

After his synesthetic-like scene of emerging into light, which dispelled the dark (accompanied by otherworldly music), *Nikoli* recounts a classic NDE sequence that largely inspired the perinatal model of NDEs, with the sound and transportation being especially DMT-esque: “An opening. I was no longer looking at the slowly spinning light at all, but through it… I began to move up. Fast. There was a whooshing sound, and in a flash I went through the opening and found myself in a completely new world. The strangest, and most beautiful world I'd ever seen. Brilliant, vibrant, ecstatic, stunning…* I felt like I was being born. Not reborn, or born again. Just… born”* [Italics added].

After elucidating his *Core* experience, encompassing darkness suffused with light, he continues: “My situation was…*akin to that of a fetus in a womb… with the silent partner of a placenta, which nourishes it and mediates its relationship to the everywhere present, yet… invisible mother* [Italics added]. In this case, the “mother” was God, the Creator, the Source… This Being was so close, there seemed to be no difference at all between God and myself.”

Similarly, the following two excerpts illustrate what could be interpreted as a *shamanic rebirth* (Winkelman, 2002, 2010) or the *Deus Ex Machina* (an ancient motif displaying divine salvation or supernatural rescue):

In this initial primitive world, *Nikoli* becomes aware of a smell, “like feces, a little like blood, and a little like vomit… of biological death,” then reports that suddenly something emerges from the dark and that he would “never be able to… come anywhere close to describing how beautiful [this entity] was.” Incidentally, St John (2015) expands on contemporary and aboriginal Australian experiences of DMT often plunging the experiencer into such grotesque, blood/vomit/skeleton permeated landscapes in such a context as the “shamanic ordeal.”

The following rebirth imagery, especially, is also entangled with the above lucid dream model, where deliberate attention or intention may drive a significant shift in (seemingly) external experience:

“I would fall and tumble out of that sanctum sanctorum, that *Core* realm of pure oneness with the divine back down to the *Earthworm's Eye-view*, and it was only by remembering the musical notes of the *Melody* that I could then conjure up the portals that… enabled me to re-ascend… into that *Gateway* valley… and then witnessing those angelic choirs above… served as a portal to higher levels.”

#### Cortical disinhibition and the anarchic brain

The below excerpts from the subject exemplify that their view on the NDE's potential induction via such endogenous chemicals is not purely predicated on the similarities of their phenomenology, but upon more subjective opining on the available neuroscience, leading to discussions of philosophy of mind. Finally, a more nuanced, novel neural approach is taken in the analysis, but with certain caveats which themselves then provoke the resurfacing of the challenges of the origin of consciousness.

“[The neocortex] is a (sic) 6 layered system, and even disrupting the superficial two layers is enough to destroy the functional integrity of the system because the superficial layers are very important at (sic) the integration across the neocortex. The deeper [subcortical] structures are more about relationships between that part of the neocortex, the thalamus and [themselves].”

This former statement points to the inherent lateral interwiring within the columnar architecture of the neocortex, where significant damage to the surface laminae interrupts the functioning of the lower ones. The latter comment implies that if indeed these neocortical laminae are more fully disrupted, this itself prevents the full functioning of the subcortical areas (e.g., the thalamus and the basal ganglia), given their reliance on the relationship with the neocortex. These sentiments are repeated in an appendix to *Nikoli'*s original documentation of his NDE. If this is accurate, which may be signified by the excessive meningeal enhancement (inflammation) and sulci obscuration by purulent (puss-filled) CSF, as well as pin-point and unresponsive pupils suggesting brainstem damage (Khanna et al., 2018). Then, this presents a challenge to any sufficient neural explanatory framework. Given the lack of detailed and direct enough data to confirm this, however, other models, such as that delineated at length later in this section or psychedelic action on the cortico-striatal-thalamic-cortical loops (Vollenweider and Smallridge, 2022), involving higher-order reception, such as frontal, instead allow fuller expression in lower-order systems.

“The important thing to keep in mind here is as a materialist neurosurgeon like I was before my coma, you have a certain set of assumptions [which]… can be your undoing… as if the brain creates consciousness. Whereas, if you simply move away from that one step and say, well the brain's not creating consciousness, but it is serving as a filter, it is limiting primordial consciousness and allowing it to express in a here and a now and sense of self… consciousness is something that exists, and what we're looking at is mechanisms to detach it from the here and now, and what I would say is a much more fundamental way to detach your consciousness from the here and now has to do with manipulations at that *lower brainstem level*… And when we do things up in the neocortex say with serotonin 2A interactive drugs, then we're altering the filter function, and it's been known for ages that altering the filter can alter the residual ‘what we experience as consciousness.' But in many ways we're talking about traversing the veil and getting to consciousness on the other side of that filtering mechanism [i.e., the brain].”

In such contemplation, the participant appears to express a non-materialist perspective. That is, such damage to his neocortex allowed a total detachment of his consciousness to experience, which greatly expanded awareness in a way that is comparable to agonism at the serotonin (5HT) 2A cortical receptors, which also results in a similar reversal of the filtration of mind and where an interruption at the more basic brainstem level instead represents (somehow) a yet even more efficient mode of achieving this (presumably where total brainstem disruption, i.e., brain death, is the most fundamental liberation). This brings up a diametric opposition of ontologies, yet it is predicated on the same neuroscientific matter of neural mechanisms, i.e., higher cortical or 5HT-2A-mediated disinhibition of mental content. That is, *Nikoli*'s own view is equivalent to a transcendentalist “transmission” theory of the brain in which consciousness is filtered from a non-material source, and the alternative is the conventional model in which consciousness is filtered while still being intrinsically brain-generated.

In this frame, what could amount to a debate on the “hard problem” of consciousness, i.e., from *where* does consciousness originate, either the brain itself or another undefined trans-material source, would arise if not for a slightly more nuanced appraisal of the neural mechanisms. Generally, any disruption of the filtering mechanisms leads to, and any resultant “heightened consciousness” is a derivative of, the *disinhibition* of deeper, normally constrained neural networks. More specifically, the “anarchic brain” or REBUS (Relaxed Beliefs under Psychedelics) model (Carhart-Harris and Friston, 2019) attempts to unify the entropic brain theory based on psychedelic neuroscience (Carhart-Harris et al., 2014; Carhart-Harris, 2018) and the free energy principle based on the brain as a predictive processor. The model clarifies that when psychedelics agonize the layer 5 pyramidal neurons' 5HT-2A receptors, the brain undergoes a disintegration of the Default Mode Network (DMN), which is itself a higher-cortical connector hub, thus leading to a release of lower, which is otherwise inhibited circuits, as well as the desegregation of otherwise disconnected non-local networks. Computationally, such higher-level cortical tissues encode top-down “priors,” representing prior knowledge of the world as learned through developmental history and generating the brain's internal model of the environment. These priors serve to inhibit bottom-up, incoming sensory data in the case where the generative model is consistent with such extrinsic information. Where these sources are incongruent, the bottom-up data represent “errors,” which are not inhibited and serve to update the internal model. Under 5HT-2A agonism, like psychedelics, however, the respective weighting between these top-down and bottom-up signals is fundamentally disrupted, and the brain is less capable of predicting or explaining the cause of one's immediate experience, and so the error signals, tantamount to raw sensory information, flood one's consciousness. As such, Huxley's “reducing valve” of the nervous system is reversed, and the “mind at large” is liberated.

In this way, if certain manifest overlaps with the NDE and the 5MeO or other psychedelic experiences do not directly argue for the release of such neurochemicals, alternative triggers that converge on the same or similar ultimate neural mechanism are still entirely possible. One novel conclusion of this study points to the unique etiology of conditions such as meningoencephalitis of the higher laminae of the neocortex, possibly constituting such a convergent mechanism. That is, in effect, it may simulate similar downstream neural activity as that underpinning classical serotonergic psychedelics' neural mechanism of action. As such, the top-down cortical disinhibition of major high-level cortical nodes in the psychedelic instance, triggered by 5HT-2A agonism of layer 5 pyramidal cells, and in the meningoencephalitis case, triggered by damage to neocortical laminae including such a layer, would lead to the release of bottom-up intrinsic cortical/subcortical information, thereby “passing up” the neural hierarchy and finally converging on a massive expansion of conscious experience. Alternatively expressed, top-layer impairments in the cortical hierarchy interrupt their capacity to construct and downwardly transmit accurate predictions, thus amounting to failures in predicting and suppressing error signals (sensory input). This then enables the errors' unrestrained propagation up the hierarchy and increases sensitivity to extrinsic or intrinsic informational input. The cortex's natural response here to minimize the errors via updating the generative world model becomes futile, instead leading to a more entropic neural and fluid phenomenal state, which is precisely like the worlds inhabited by those after ingesting psychedelic substances (moreover, in reference to *Nikoli's* preference for brainstem manipulation for such consciousness expansion, theoretically, stimulation at such deeper sites as the brainstem could mimic any intrinsic activity release from otherwise inhibitive higher, inhibiting cortices).

This type of model, where impairment of more evolutionarily recent cortices results in an (initially counter-intuitive) elevation in conscious experience, echoes similar reports in the literature. This includes the finding that those with traumatic brain injury with lesions specific to the middle-superior temporal, but especially the dorsolateral prefrontal cortex, scored significantly higher than controls on mysticism measures, underscoring the causal role of executive regions in downregulating mystical experience (Cristofori et al., 2016). Additionally, resection during brain tumor surgery of the inferior posterior parietal lobes (constituting the lateral node of the DMN), including the inferior parietal lobule (left hemisphere) and the angular gyrus (right hemisphere), increased the reporting of self-transcendence, involving constructs such as unity, space, and timelessness (Urgesi et al., 2010). This is also consistent with the widely popularized case of Bolte-Taylor's “stroke of insight,” where an infarction disabling much of her left hemisphere (normally exerting a dominant inhibitory effect on the right) leads to a profound mystical experience of selfless unity (Taylor, 2009). Similarly, many reports of acquired savant syndrome, wherein after traumatic brain injury or dementias (e.g., frontotemporal), some individuals present with specially developed cognitive or artistic capacities, are likely also relatable to the disinhibition of lower-level structures due to the inactivation of higher, inhibitive zones such as the prefrontal cortex (Takahata and Kato, 2008). This itself is equally compatible with the predictive coding frame, where such disinhibition is equivalent to disrupted high-level predictions, leading to increased sensory data/error (Gallimore, *personal communication, 1st March 2022*). Such studies also highlight that the encephalitis of this case is only one of these other exemplary conditions in which the above disinhibition processes occur and, similarly, that these processes may be a core undergirding mechanism for NDEs caused by brain trauma (e.g., Hou et al., 2013) and virtually all other NDEs whose aitiology eventually converges on neural anoxia; hence, the near-universality of phenomenological features across NDEs.

However, this proposition, as applied to the case of *Nikoli'*s NDE, is not without its caveats, such as the possibility of at least some component of the neocortex still being necessary for the reception of any released intrinsic activity, as well as that pointed out by *Nikoli* that even superficial damage to the top-most neocortical laminae may undermine their entire functional integrity. As the neocortical damage in this case appears highly diffuse and non-selective, there may not be the necessary preservation of minimal neocortical function for the construction and experience of any type of inner world (Gallimore, *personal communication, 1st March 2022*). This possibility, compounded by the fact that any deeper subcortical zones, despite their preservation, are not computationally sufficient to account for the patently exceedingly elaborate phenomenology of the present NDE, makes for a notable challenge for a fully explanatory, neurally mechanistic model. While other authors (Khanna et al., 2018; Greyson, 2021) have argued that the damning medical data, such as the *structural* CT scan report, is sufficient to claim the inadequacy of the brain to drive this particular NDE, the glaring lack of any *functional* imaging tools (EEG/PET/fMRI) employed during *Nikoli*'s hospital stay precludes the full legitimacy of this statement (Michael, 2021).

#### Other neural mechanisms

Further to the putative neural contributions to the near-death experience, not noted in previous reports on this participant's NDE (e.g., Khanna et al., 2018), is the possible role of the seizure with which the patient first presented upon entry into the emergency room. All of the complex partial, absence, and generalized effects demonstrated by the present case have been shown by Danielson et al. (2011) to co-occur with significant reductions in the activity and functioning of the default mode network (DMN) via discharges that result in the inhibition of arousal-promoting nuclei that otherwise help to drive the DMN. The disintegration of the DMN and concomitant network desegregation under the influence of psychedelics, as discussed above, are considered to be the primary functional connectivity changes undergirding the drugs' psychoactive profile, with initial evidence for this in the form of reduced oxygen perfusion in the DMN under psilocybin (Carhart-Harris et al., 2012). As such, in line with the present analysis' argument for the substantial phenomenological resonances between the NDE and 5MeO and other psychedelic states, although no direct evidence has been provided for similar alterations in DMN activity during near-death, it is highly plausible due to the said resonances. The generalized seizure, presented by *Nikoli* at the very onset of his meningoencephalitis illness, may feasibly be yet another key neurobiological correlate, for he subsequently reported an NDE. The fact that such epileptic activity may be highly associated with NDE-like states is suggested by an overlap between ictal and NDE (Blackmore, 1998; Hoepner et al., 2013; Greyson et al., 2014) or other mystical phenomenology (Coles, 2013; King et al., 2019) and the physiology of seizure, especially temporal activity, being linked to reporting NDEs (Britton and Bootzin, 2004) or other florid dreamlike states (Carhart-Harris, 2007).

However, his NDE has been “placed” instead on hospital days 1–5 when GCS scores indicated a deep coma, ranging from 6 to 7, as he accurately reported “bedside visits from non-family members from an out-of-body perspective.” However, this is not entirely logical, as this perceptual report can only temporally situate the out-of-body perceptions of his physical surroundings, which may be entirely dislocated from his wider NDE; indeed, this is suggested by his not reporting an out-of-body experience (OBE) at the onset of his NDE, which is otherwise characteristic of most NDEs (Charland-Verville et al., 2014). As such, the NDE could have occurred in a less compromised brain state. Nevertheless, the reporting of such anomalous phenomena as these reputedly veridical OBEs as part of his NDE account, which also includes an alleged “peak in Darien” experience, in which he encountered a presence he only subsequently identified as an unknown deceased family member, gestures toward parapsychological events that are not accommodated by the present neural models and so also toward the need for further investigations of such challenging features (beyond the scope of the present article).

Additionally, the emphases placed by *Nikoli* on so-called “counterfactuals,” which occurred mainly in his NDE but also in the 5MeO trip, where he reported witnessing a vast tapestry representing alternative life trajectories, including future pathways such as those based on a range of prior decisions, may also have some neurobiological explanation. Van Hoeck et al. (2013) reported that compared to the episodic memory of the negative past and the imagining of positive future events, counterfactual cognition (constructing different and better future outcomes from past events) was associated with more extensive activation of the medial temporal lobe (e.g., core memory circuits), the medial prefrontal cortex (e.g., theory of mind), as well as additional recruitment of the bilateral inferior parietal lobes, which include the angular gyrus (e.g., body schema, attention, declarative memory, and language). The latter two regions, incidentally, directly overlap with the nodes of the DMN, and the former is intrinsically associated. The precise mechanisms underlying *Nikoli's* subjective visualization of the said counterfactuals, in the highly intricate form described, cannot be readily accounted for. However, the above-delineated neuroimaging data, compounded by the quintessential effects of psychedelics being to “reveal the mind,” can provide a working framework. An analogy to this may be the kaleidoscopic hallucinatory displays, which are a product of occipital disinhibition, in which you are “basically seeing your own visual architecture” (Cowan, 2013).

## Discussion

### Core comparability resides in mystical experience

Taken together, various similarities can be observed between the participant's original, naturally occurring NDE and the psychoactive experience elicited by 5MeO-DMT, such as ego dissolution, unitive experience, cosmic love, ineffability, and most evidentially from the above analysis, transcendence of time and space, especially the experience of “counterfactuals” (linear time transforming into an expanded view of alternate life trajectories), by which hyperdimensionality may also be encompassed. This suggests that NDE and 5MeO share commonalities in the domains of the mystical experience, particularly in five of its categorized dimensions (with cosmic love resonating with the “bliss” factor (MacLean et al., 2012).

Similarly, while numerous themes of *Nikoli's* NDE were identified as being different from his own 5MeO experience, they are still either characteristic of, or sufficiently common in, other psychedelic experiences, especially *N,N-*DMT, and some were also reported with 5MeO, including the void or profound learning. These themes include encounters with divine or menacing beings, otherworldly experiences often resembling earth, voids, synesthesia, life reviews, encounters with deceased loved ones, thresholds, and learning.

### Elaboration on responses by previous authors

Considering this, Harris (2014)—prior to *Nikoli*'s experimentation with 5-MeO-DMT—represents a previous comparison of the subjects' NDE with an analogous DMT state. He details a similar argument that the imagery in his original NDE report, contrary to *Nikoli's* protestation of it “not being in the same ballpark” of a psychedelic experience, is “the stitching on the same ball” and perfectly reproducible by an *N,N-*DMT trip. Although, to be more nuanced, as indicated by the present analysis, the more dualistic (*I-It*) experiences surrounding *Nikoli*'s *Gateway* realm are more inducible via such DMT trips. However, the special evocation of the NDE by his 5MeO experience, i.e., the profound mystical and non-dual (*I-thou*) dimensions, especially in the *Core* realm, may suggest that endogenous 5MeO could contribute specifically to these subjective motifs, leading to a model in which different endogenous chemicals have differential contributions to separate phenomenological domains.

Harris (2014) also made an important observation that, though *Nikoli* states in his book that loss of cortical activity is “clear from critical global involvement documented by CT scans,” activity can only be appropriately determined by functional vs. structural imaging (Michael, 2021). However, he also cites that 50–70% of cortical activity remains in comatose patients, where although half of the normative cortical activity may remain, it is crucially the processes of network co-activation, complexity, and information integration that are largely considered pivotal for the sustaining of consciousness (Seth et al., 2011), which may well have been insufficiently maintained given the encephalitic damage. Despite this, such functional data is not available, and in light of the hypotheses speculated in the *Anarchic Brain* segment above, the reduction of high-level networks may indeed elevate such lower hierarchical entropy and integration, in turn mediating high-intensity conscious experience, as seen under psychedelics (Carhart-Harris and Friston, 2019). Moreover, in countering *Nikoli*'s argument that the cortex is what is assumed to be requisite for consciousness, and yet he was disabled during his NDE, Harris reminds us that “no one thinks that consciousness is just a matter of the cortex;” however, the “content” of consciousness, such as the baroque, multisensorial, and narrativized NDE *Nikoli* reports, is considered to rest upon cortical integrity, while “wakefulness” itself is mediated by the brainstem nuclei (Martial et al., 2020).

Returning to Harris's (2014) appraisal, his point that endogenous DMT requires only a few minutes of brain activity to engender an “eternal experience” is salient and especially pertinent because the fact that anybody remembers their NDE necessarily suggests that neural structures are required for short-term memory formation and long-term consolidation. Presumably, the structures that are active during retrieval that mirror the original structures active at encoding must have been at least sufficiently functional during the critical period for the subsequent re-recruitment of those same memory traces during recall. This presents a significant challenge to any notion of non-local consciousness during the NDE, given the problem of a lack of encoding substrates despite eventual retrieval, supporting the “shut down” or “reboot” timing for the experience.

### Proposed mechanistic models

Even while accepting these striking similarities, this in itself does not immediately justify a model in which the dying brain produces endogenous psychedelics (which 5-MeO-DMT and *N,N-*DMT both are), thus accounting for the phenomenological overlay. As discussed above, phenomenology also remarkably echoes lucid dreams as well as perinatal regression (where the latter mirrors only certain features, such as primitivity, boundarylessness, and rebirth). All the psychedelic, dream, and perinatal perspectives, however, are not mutually exclusive, given psychedelics and lucid dreams' ability to partially reproduce each other and psychedelics' induction of perinatal experience.

Importantly, however, while the initial triggers may all be independent, the end-point neural mechanisms may be highly similar between each of them. This is especially concerning the already elaborated concept that endo-psychedelics need not be implicated in the near-death state, or in this case, a meningoencephalitis-induced coma, as they may both converge on the down-stream mechanism of a release of suppressed, intrinsic bottom-up information.

However, it should be noted that there are caveats to this theory, as a sufficiently intact neocortex may still be required to process the released data and preserve the function of deep-brain subcortical regions.

### Psychedelic experiential repertoire

Despite the similarities in phenomenology, the participant was insistent that his NDE and psychedelic experiences were insufficiently similar, and thus, endogenous psychedelics did not play a role in the induction of his NDE. This is reflected in his very low quantitative appraisal of 2/10 for both similarity and likely psychedelic induction. This opinion may be in part due to the lack of adequate personal experience of, as well as overall familiarity with, psychedelics (*Nikoli* himself admits only one other less significant experience with *N,N-*DMT and some lower-dose LSD and mescaline trips in his adolescence). Thus, he may be less equipped to draw the extant and extensive parallels in the same way that the subculture of the psychedelic community *is*, which has developed a nuanced vocabulary to parse these transcendent states.

### Implications for consciousness

Ultimately, despite this novel neural model of the neocortical damage simulating the expanded consciousness resulting from psychedelic action, this is not necessarily incongruent with a transcendentalist interpretation of this striking near-death experience. This is a “neurological” conception (Strassman, 2014), which is consistent with the transmission theory of the brain and consciousness and may be complemented by another more nuanced neurobiological perspective. While this proposed model does constrain the need to invoke some trans-material source of consciousness, the “hard problem” remains, given the apparent gap between the physical substrate, even the computational mechanisms of the brain and subjective awareness. However, the free energy principle attempts to bridge this gap (Solms, 2021), for instance, via the predictive processing of interoceptive input (Solms and Friston, 2018), which is not necessarily reliant on cortical but deep brainstem mechanisms regulating affective states, where the modeling of “feeling” may instead be pivotal for engendering conscious experience. Many other theories of consciousness applied to psychedelic action also approach the closing of this gap, such as cortico-thalamo-cortical loops (Preller et al., 2019) and cortico-claustrum connectivity (Stiefel et al., 2014; Barrett et al., 2020). However, this is critiqued as helping to explain only the contents (the “easy problem”) vs. the appearance of phenomenal consciousness (Yaden et al., 2021).

### Limitations

Finally, one limitation of the present study is that the participant has limited prior experience with psychedelic drugs. This may have resulted in a lack of the conceptual repertoire necessary to recognize the overlap between his own and, indeed, others' NDE and 5MeO/psychedelic experiences and, therefore, the potential inducibility of NDEs by endogenous psychoactive chemicals. Importantly, this may highlight the possibility of inexperienced individuals understating the role of such chemicals in driving near-death or similar spontaneous states, which is a tendency of many authors or commentators in the field of near-death studies or its community. Future studies may thus benefit from recruiting those with a greater experiential repertoire. In spite of this limitation, the interpretations *Nikoli* provides in understating this possibility should be recognized, where he emphasizes the shallowness of his 5MeO trip compared to the profundity of his NDE. Another limitation, not unrelated, may be any ideological biases that *Nikoli* may hold that may lead him to such a conclusion. This is alluded to in many of his above statements, wherein, as a direct transformation in metaphysical beliefs after his NDE (as also identified after psychedelic experiences, Timmermann et al., 2021; Nayak and Griffiths, 2022), he eventually rejected mechanistic neuroscience in favor of a transcendentalist/post-physicalist paradigm, thereby driving a potential prejudice against a reductive account of his NDE, as implied, yet not entirely mandated, by a neuropharmacological model. These limitations are also partly a side effect of employing a single case study to delineate the psychedelic-NDE relationship, which similarly inherently limits the range of qualitative content generated, and as such, further studies are warranted of either a between-subjects comparison of the different experience types (Michael et al., in review) or within-subjects amongst those reporting both (as herein; Michael et al., in review). The scant population, however, in this latter camp was one reason for the single case study.

## Data availability statement

The raw data supporting the conclusions of this article will be made available by the authors, without undue reservation.

## Ethics statement

The studies involving human participants were reviewed and approved by University of Greenwich Research Ethics Committee (Ref. 18.5.5.17). The patients/participants provided their written informed consent to participate in this study. Written informed consent was obtained from the individual(s) for the publication of any potentially identifiable images or data included in this article.

## Author contributions

PM: conceptualization, data curation, formal analysis, and writing the original draft. PM, DL, and OR: methodology and review and editing. DL and OR: supervision. PM and DL: funding acquisition. All authors contributed to the article and approved the submitted version.
