# Supplementary material for: This is your brain on death: a comparative analysis of a near-death experience and subsequent 5-Methoxy-DMT experience
Source: Front Psychol. 2023 Jun 29;14:1083361. doi: 10.3389/fpsyg.2023.1083361 (PMC10345338; doi:10.3389/fpsyg.2023.1083361)
Supplement: Supplementary file 1 [file Table_1.DOCX]

**Supplementary Material**

**SM 1.**

**Example questions from semi-structured interview**

**(including paraphrasing of participant’s prior answers)**

*Please describe in as much detail as possible your experience with 5-MeO-DMT*

*…in your narrative [of] your NDE there was a rich visual, multisensory component… so was there any kind of sensorial or visual experiential structure as well [in your 5-MeO]?*

*It was very interesting you said that having had the NDE, then latterly the…5MeO, those experiences might have had some echoes of the NDE sheerly because you had the NDE in the first instance…[is] that fair to say?*

*So it may not be the quintessential content or essence of these experiences which differ fundamentally, but maybe the approach to them, maybe even ritual and integration around them could actually be the determining factor as to how close they come together?*

*Can I ask for an elaboration of what you refer to as the ‘counterfactual’ which was quite evident in your NDE, this view you can have as your higher soul where the linear structure of time is dissolved away, and what does that mean?*

*So you think there’s a much deeper dimension of the personal, what’s reflective of your entire life on earth within the NDE... However, with…the 5MeO, there’s…this sense of profound unity and oneness…and has much less of this personal component?*

*I [also] wonder what your comments would be in terms of the threshold of no return that comes up with NDEs, and it doesn’t seem to be that prevalent with DMT?*

*Was there any memory of transition from these personal aspects like the review, toward this transcendent deep time space?*

*…you tried to recapitulate aspects of your NDE with ‘sacred acoustics’ which might be targeting these deeper brain structures, the reticular nucleus etc – you think that reticular nucleus may be shared in terms of your trying to get back [to the NDE] and the NDE itself? Do you think DMT have had a role in disturbing the reticular formation in your NDE?*

*You mentioned there some classic psychedelic fractal type tiles, so that echoes…Sam Harris, when he says your experience looks exactly like a DMT experience – but the key work is “look”? Where [you’re suggesting that] those deeper ineffable experiences and aftershocks are fundamentally different?*

*So you had no prior real conviction of understanding of reincarnation before your NDE, but within the NDE there was this compelling understanding of that being a necessary component of the universe?... did you have anything of that nature in your [5-MeO-DMT] experience?*
